# Sexual minority (LGBQ+) women’s experiences of accessing addiction services: a qualitative study

**DOI:** 10.3389/fpubh.2026.1784134

**Published:** 2026-05-14

**Authors:** Miriam Hillyard, Jamie Hakim, Colin Drummond, Katharine Rimes, Emmert Roberts

**Affiliations:** 1 National Addiction Centre, Institute of Psychiatry, Psychology and Neuroscience, King’s College London, London, United Kingdom; 2 Department of Culture, Media, and Creative Industries, King’s College London, London, United Kingdom; 3 School of Psychology, Portland Square, University of Plymouth, Plymouth, United Kingdom; 4 South London and Maudsley NHS Foundation Trust, London, United Kingdom

**Keywords:** addiction, bisexual, gay, lesbian, LGBTQ, queer/questioning, services, trans

## Abstract

**Introduction:**

Lesbian, gay, bisexual, queer and other non-heterosexual (sexual minority) women experience significant health inequities in comparison with heterosexual women. Both drug and alcohol use, and addiction to drugs and alcohol, are more prevalent for this group. They may also be more likely to underutilise addiction healthcare services in comparison to sexual minority men.

**Aims:**

We aimed to understand sexual minority women’s experiences of accessing addiction treatment services in England, UK; what prevented them from doing so if they did not; and what would have facilitated service access.

**Methods:**

A qualitative study was performed, using reflexive thematic analysis of 21 semi-structured interviews. Interviewees were self-identified sexual minority women, who all had experience of harmful or dependent use of drugs and/or alcohol.

**Results:**

A diverse group of sexual minority women (including cisgender and transgender women) were recruited. Few had accessed addiction treatment services, but those who had reported experiences of inadequate, fragmented, or disrupted care, with occasional positive experiences of acceptance and empowerment. Barriers to access included explicit homophobia and discrimination, fearing or anticipating stigma, and reluctance to approach services or label drug/alcohol use as problematic. Potential facilitators to access and engagement in treatment included being listened to and believed, having sexuality acknowledged and accepted, and being supported by LGBTQ+ peers or allies. Services could act either as sources or buffers of minority stress, but findings highlighted the impact of problematic structural and organisational factors.

**Discussion:**

Services and health professionals wishing to better help sexual minority women with addiction problems need to understand the specific needs of this marginalised group, and how these are affected by their other intersecting identities. Sexual minority women may also benefit from specific targeted interventions addressing barriers and facilitators to accessing addiction services.

## 1 Introduction

Lesbian, gay, bisexual, queer, and other non-heterosexual people may be described collectively as belonging to a ‘sexual minority’. Internationally, sexual minority people are acknowledged to experience significant inequities in health and wellbeing compared with their heterosexual counterparts (1–3). Both drug and alcohol use, and addiction to drugs and alcohol, are more prevalent for sexual minority than heterosexual people (4–8). These disparities may be particularly pronounced for sexual minority women, who appear to have higher rates of drug use, drug use disorders, and heavy episodic drinking than sexual minority men (9–11). Women with intersecting minoritised identities, such as being transgender, belonging to a racial/ethnic minority, or having low socioeconomic status may experience even worse disparities (12, 13). There are also within-group disparities, with bisexual women having elevated rates of drug and alcohol use, and use disorders, compared with lesbian women (9, 14, 15)^1^.

There are several underlying explanatory mechanisms for these disparities. The effects of minority stress and chronic social adversity are well-documented (16–18), and sexual minority women experience higher rates of exposure to trauma and victimisation across the life course than heterosexual women (19, 20). There is evidence of increased risk of childhood sexual and emotional abuse, physical parental violence, and peer victimisation in comparison with heterosexual youth (21, 22). Mental health problems and problematic drug/alcohol use are common consequences of such trauma (19, 23–25). Sexual minority women may also experience family rejection on the basis of their sexual orientation, and therefore may lack social support compared with heterosexual women (26), which is often key in addiction recovery (27, 28).

Accessing appropriate and affirmative healthcare is often challenging for sexual minority women. In general, women with drug and alcohol problems underutilise specialist addiction treatment compared with men (29). The reasons for this may include greater stigma, concerns about losing child custody or parental rights, and other competing family/caring demands (30). Concerns about re-traumatisation (e.g., having to explain a trauma history repeatedly) may also represent a barrier to treatment (31, 32). Studies examining sexual minority people’s perceived barriers to accessing general health services point to poor provider knowledge, heterosexism, or outright discrimination from staff or services (33–36). A systematic review by the authors about barriers and facilitators to addiction treatment for sexual minority people (37) showed a significant under-representation of women in this body of research.

The standard psychomedical discourse of LGBTQ+ illness has historically conceptualised LGBTQ+ people as ‘vulnerable’ subjects, ‘at risk’ from negative health outcomes related to minority stress (16, 38). In this model, “distal” or environmental stressors related to minority status (such as homophobic violence) may be internalised, creating “proximal” stressors (such as identity concealment or expectation of rejection). The negative health outcomes created by these stressors can be interrupted by coping factors, such as connection to LGBTQ+ community. Although minority stress theory has been instrumental in shifting explanations of health disparities away from individual pathology and towards social causation, it tends to locate the experience and management of risk within the individual, rather than in the institutional or structural conditions that produce marginalisation (39). More recent scholarship has called upon research to investigate the interconnecting social, cultural, political, and economic factors that produce states of ill-health, focusing on the lived experience and subjectivities of LGBTQ+ people (40–42).

Rather than sexual minority women being framed as inherently ‘damaged’ (43), it may be the case that the health systems intended to serve them do not meet their needs (44, 45), or that overarching systems of power interact to produce particular configurations of advantage and disadvantage, that shape both drug/alcohol use and service interactions (46). Qualitative work indicates that alcohol and drug use for sexual minority women may serve functions that are invisible or unintelligible within conventional healthcare: including positive roles in identity formation (47, 48), community connectedness (49, 50) active social resistance to a dominant heterosexual majority (51), a therapeutic activity (52), or a way to express and codify queer sexuality (53). Recognising this context has implications for the design of services and public health policies that engage with the lived realities of sexual minority women’s lives.

## 2 Aims

Building on existing evidence from the United Kingdom (UK) on LGBTQ+ substance use and help-seeking (54–57), this study forms part of a larger qualitative project exploring sexual minority women’s experiences of addiction to drugs and/or alcohol. We investigated their routes to addiction recovery via both formal addiction treatment services, and more informal support (e.g., peers or mutual aid groups). The wider study has both descriptive and analytic aims, but in this paper, we speak specifically to the relative lack of qualitative research examining sexual minority women’s experiences of accessing specialist addiction services in England.

Here, we aim to answer three research questions:

1. What are the experiences of sexual minority women accessing specialist addiction treatment services?
2. If they did not access services (or struggled to remain in services they had accessed), what were the barriers to doing so?
3. What would have made accessing services easier or more possible?

## 3 Methods

### 3.1 Study design

Wanting to illuminate participants’ social realities, a qualitative study design was chosen. This allowed for the collection of rich, narrative data, facilitating detailed exploration of sexual minority women’s lived experiences. What counts as ‘problematic’ drug and alcohol use may be mediated by cultural scripts, community norms, stigma processes, and historically specific understandings of sexuality (58–60), which are hard to capture in quantitative surveys. We also aimed to understand the sense that sexual minority women make of their interactions with institutions, and decisions about whether to seek help from them. We sought to explore nuanced meanings, emotions, and contextual dynamics around these decisions, foregrounding the perspectives of sexual minority women themselves. Individual interviews were chosen to enhance relational trust and disclosure, understanding that speaking about sensitive or traumatic experiences may be more challenging in a group, and to allow the interviewer-interviewee dyad to focus on individual life histories in depth. This approach also allowed for participant diversity (e.g., gender identity, sexuality, or ethnic/racial identity) to be explored.

### 3.2 Recruitment

The study received approval from the King’s College London Research Ethics Committee (REC number: HR/DP-21/22–25946). Potential study participants were recruited via paid adverts and posts placed on social media (Facebook/Instagram), through relevant LGBTQ+/sexology and university mailing lists, and via word of mouth. This allowed initial convenience sampling followed by more purposive sampling (e.g., altering subsequent adverts to ensure a broad sample based on gender identity and ethnic/racial identity). All potential participants were directed to an online information sheet about the study. After providing an email address, potential participants were contacted by the lead author (MH) to confirm their eligibility. All participants provided written consent prior to their interviews. 44 potential participants were contacted, of whom 21 agreed to an interview. No participants dropped out after initially agreeing to an interview, or asked to withdraw following their interviews; and each participant was interviewed once only.

### 3.3 Inclusion criteria

Participants had to be ≥18 years old, able to conduct an interview in English, and a UK citizen or ordinarily resident in the UK in order to be included in the study. They had to self-identify as a sexual minority (i.e., non-heterosexual) person, and also as a woman. For the purposes of our study, our definition of women was inclusive, and we explicitly noted in our recruitment information that we were seeking to speak to cisgender and transgender women. Participants also had to have past or current experience of harmful or dependent drug and/or alcohol use. We chose to trust participants’ self-report of this, and they were asked to describe in detail their previous experiences with drugs and/or alcohol during their research interviews. As such, MH used her clinical experience to broadly confirm their self-reported experiences, although she did not conduct a formal clinical addiction diagnostic assessment. As we were seeking to understand barriers to accessing addiction services, there was no criterion that participants had previously accessed or tried to access services.

We defined specialist addiction treatment/services as consulting with a healthcare professional (such as a doctor, nurse, psychologist, drug keyworker etc.) who has specialist clinical training in the management of addiction problems. This includes, for example: attending a drug and alcohol service, being seen by a drug/alcohol liaison team in a hospital, or receiving an addiction intervention (e.g., opioid substitution prescriptions) from a specialist General Practitioner (GP) service. It does not include, for example: receiving brief advice about drugs/alcohol, attending general GP appointments, mutual aid meetings such as Alcoholics Anonymous (AA) or Narcotics Anonymous (NA), receiving faith-based interventions delivered by religious organisations, or more informal support from family/friends (findings about which will be presented in a different paper). In England, statutory addiction treatment services are commissioned locally and provided for free at the point of access, but in practice, vary significantly in terms of service provision and funding (61). Although general mental health and talking therapy services are also available as part of the UK’s public National Health Service (NHS), these typically focus on addressing mental health concerns and usually do not directly work with addiction problems.

### 3.4 Positionality

The interviews were all conducted by MH, who is a queer cisgender woman. She is a doctor (BM BCh, MSc) who at the time of the interviews was working clinically in addiction services; a psychosexual and relationship therapist (RegCOSRT); and a PhD candidate with a background in LGBTQ+ health research. She has experience of working clinically as a doctor and therapist with sexual minority women, and also of leading or participating in various voluntary LGBTQ+ support, education, and activism projects. No participants were personally known by her before their interviews. Her sexual orientation and gender identity were shared with potential participants in the recruitment information, and before the start of each interview she gave a general statement about her motivations for conducting the research (e.g., “I wanted to do this study because of my own experiences, and what I’ve seen in my queer community”). She used her ‘insider’ status (e.g., showing familiarity with LGBTQ+ culture and terminology) to build rapport with participants during interviews (62), although at times leaning on her ‘outsider’ or ‘expert’ positioning as a clinician/researcher (e.g., explicitly naming common addiction-related patterns or experiences encountered in her clinical work). She had received specific qualitative methods training prior to conducting the interviews, and had previous experience of qualitative research (63). The wider research team mostly identify as LGBTQ+, and have expertise in both qualitative and quantitative LGBTQ+ health research and/or addiction research. JH is a Senior Lecturer in Media and Cultural Studies, CD is an addiction psychiatrist and Professor of Addiction Psychiatry, KR is a clinical psychologist and Professor of Clinical Psychology, and ER is an addiction psychiatrist and Senior Clinical Lecturer in Addiction Psychiatry.

### 3.5 Interviews

Semi-structured telephone interviews were conducted with all participants (except for one deaf participant, who was interviewed in written format via Microsoft Teams chat; responses from that participant have been edited into quotations and are presented in the same format as those from the other participants to preserve anonymity). Participants could remain anonymous if they wished (e.g., using a pseudonym). Basic demographic information (e.g., age, sexual orientation, gender identity) was collected during the interview. Participants were asked to be alone in a private indoor location for the interview, which typically lasted around an hour. They were asked to confirm they were not intoxicated at the start, and MH also used her clinical experience during the interview to assess this, with a protocol in place for ending the call if any participants disclosed intoxication. Before starting each interview, MH outlined the planned structure and gave examples of the types of questions that would be asked. She explained that participants could take a break at any time, or ask to skip any questions that they did not feel comfortable answering. She explained the limits of confidentiality, and verbal consent to the interview was re-ascertained following this introduction.

Interviews loosely followed a topic guide, although also allowed both interviewer and participant to discuss any additional information which seemed relevant. Participants were initially asked about their journey of coming to understand their sexuality and gender identity, followed by experiences with drugs and/or alcohol. If they had accessed addiction services, they were asked to describe their experiences. If they had not accessed services, they were asked what had prevented them from doing so, and how they had recovered from addiction problems (if these were historical). Finally, participants were asked to imagine what might facilitate access to existing services, and what ‘ideal’ services for them might look like. The topic guide was initially constructed based on previous published qualitative and quantitative literature, and was piloted with two sexual minority women with relevant experience (known to MH) in the development stage. This piloting resulted in amendments to the initial topic guide (e.g., adding in questions about gender presentation/conformity and LGBTQ+ community connection). A protocol was also developed in case any participants became distressed during the interview or disclosed acute risks (e.g., suicide plans). Following the interviews, participants received information about relevant addiction, mental health, and LGBTQ+ support organisations. Participants were given a £30 shopping voucher for taking part in the study.

### 3.6 Data analysis

Interviews were audio-recorded and MH also wrote notes during and after each interview. Recordings were professionally transcribed, and each transcript was checked for accuracy, and anonymised, by MH reading through whilst listening back to the audio recording. NVivo software (Lumivero, LLC; version 15) was used to assist analysis of transcripts and notes, and to organise research data. Reflexive thematic analysis (as described by Terry, Hayfield, Clarke & Braun, 2017 (64)) was chosen as the analytic method. This is a flexible, organic approach in which “researcher subjectivity [is] understood as a resource” (65) and “integral to the process of analysis” (64), allowing both inductive and deductive approaches to coding, and the generation of themes through deep and recursive immersion in the data. The researcher constructs themes as an active and interpretive process; integrating and contextualising the research data with existing theoretical frameworks, subjective viewpoints and experiences, and disciplinary knowledge. Interpretation of our data was informed theoretically by Meyer’s minority stress model (16), but we also used concepts from intersectionality to make sense of our findings (46). Firstly, MH familiarised herself with the data by reading and re-reading each interview transcript, making brief initial notes and provisional observations. Following this process, initial codes were generated, tagging both semantic (descriptive) and latent (interpretative) meanings in the participants’ words. We used both deductive and inductive coding within the transcripts, but for the sections of the interviews speaking to the research questions that this paper addresses, coding was primarily deductive. Following the coding of the data, candidate themes were constructed, understood as “patterns of shared meaning underlain by a central meaning-based concept” (64, 65). Candidate themes were generated from the data, rather than mapping on to specific questions from the interview topic guide (i.e., we proceeded from codes to themes, rather than coding for pre-specified themes). These candidate themes were reviewed, developed, and refined iteratively, before being written up (understood as a final process of analysis). Regular meetings with the wider supervisory team throughout the process helped to clarify and refine themes and address reflexivity issues. Of note, each participant was asked during their interviews which pronouns they used (e.g., she/her, they/them) and these are used where relevant in presenting the results.

## 4 Results

### 4.1 Demographics

Between March 2022 and January 2025, 21 interviews were conducted, ranging from 40 min to 89 min (mean: 54 min). Wanting to avoid potential identification of participants from small minority-within-a-minority communities, they are referred to by participant numbers, and a summary of their demographic information is provided in Table 1. Participant ages ranged from 20 to 62 years (mean: 33 years), and they described a range of gender identities, including cisgender and transgender women, and women with a more fluid gender identity. Participants used a range of terms to describe their sexuality, including bisexual, lesbian or gay woman, pansexual, queer, polyamorous, and “no label.” In terms of race/ethnicity, participants were mostly white (67% of the sample).

**Table 1:** Demographics of the interview participants (*n* = 21).

Demographics	Frequency
Age	
≤20	2
21–25	5
26–30	2
31–35	4
36–40	4
41–45	1
46–50	1
≥51	2
Self-described gender identity (some participants described more than one)	
Woman	16
Trans woman	2
Detransitioned (trans man to woman)	1
Non-binary	1
Nullo	1
“Mostly woman”	1
“Femme with masc vibes”	1
Self-described sexual orientation (some participants described more than one)	
Bisexual	10
Lesbian/gay woman	6
Pansexual	4
Queer	4
Polyamorous	1
“No label”	1
Ethnicity/racial identity	
White British	7
White Other	7
Asian or Asian British	2
Mixed Race (including people with self-described mixed Caribbean, Asian, Latin American, White, and Indigenous identities)	5
Location	
South West England	3
South East England	4
West Midlands	1
London	9
Midlands	1
North West England	1
East of England	2
Recruitment method	
Social media adverts (Facebook/Instagram)	11
LGBTQ+/sexology mailing lists	2
University mailing lists	4
Word of mouth	1
LGBTQ+ Facebook group	1

Terminology notes: ‘nullo’ has multiple meanings but typically refers to people who desire a ‘neutral’ genital appearance and/or have sought surgery to remove the external genitalia. Polyamorous typically refers to the practice of having more than one romantic relationship at a time (or a desire for this).

### 4.2 Drug and alcohol use and service use history

All participants described current or former harmful or dependent use of drugs and/or alcohol, with the vast majority having used both drugs and alcohol. Fifteen participants self-reported current or former alcohol dependence, and seven current or former drug dependence. Table 2 presents summary data on the participants’ drug/alcohol use histories. Drugs used included (in order of frequency across the study participants): cannabis, 3,4-methylenedioxymethamphetamine (MDMA), cocaine, psilocybin (magic mushrooms), amphetamines (speed), lysergic acid diethylamide (LSD), ketamine, heroin, prescription opioids, benzodiazepines, dimethyltryptamine (DMT), fentanyl, poppers, kratom, mephedrone, and 2C-B.

**Table 2:** Combinations of drug/alcohol problems described by study participants (*n* = 21).

Type of self-reported drug/alcohol problem (current or former)	Frequency
Drug dependence and alcohol dependence	4
Drug dependence and harmful use of alcohol	3
Alcohol dependence and harmful use of drugs	4
Alcohol dependence and occasional use of drugs	6
Drug dependence and occasional use of alcohol	1
Harmful use of alcohol and harmful use of drugs	1
Harmful use of alcohol and occasional use of drugs	1
Alcohol dependence and never tried drugs	1

Only a small minority (5 out of 21) of the study participants had ever accessed specialist addiction treatment or services, of whom 1 had been immediately deemed ineligible and turned away from the service. Some participants had attempted to seek help from other non-specialist health services. Nine out of 21 had spoken to a GP or GP practice nurse about drug/alcohol problems, and 6 out of 21 had been in contact with emergency or acute hospital services for drug or alcohol-related reasons (e.g., following an overdose). Four participants reported disclosing drug/alcohol problems in the context of being seen by general statutory mental health or talking therapy services. Three out of 21 participants reported never having been in contact with any primary or secondary care health services to speak about drugs/alcohol. However, 20 out of 21 participants said more informal or non-statutory support had helped them recover from their drug/alcohol problems. This included support from friends, family, partners, LGBTQ+ and non-LGBTQ+ peers, online groups/forums, mutual aid groups such as AA or NA, teaching staff at schools or universities, and private psychotherapists. Of note, 3 out of 21 participants had received privately provided sexuality or gender conversion ‘therapy’ aimed at making them heterosexual or cisgender.

In line with our research aims, we will present themes relating to 1. the experiences of participants who did access specialist addiction treatment, 2. barriers to accessing or remaining engaged in addiction treatment and 3. what might have facilitated access to services (or made them better for those who did access them).

### 4.3 Themes

Table 3 summarises the themes generated for each of the research aims.

**Table 3:** Summary table of the themes relating to the three research questions of the paper.

Questions	What are sexual minority women’s experiences of accessing specialist addiction treatment?	What are the barriers to accessing or remaining engaged in addiction treatment?	What are the facilitators to accessing or remaining engaged in addiction treatment?
Themes	Experiencing inadequate care	Explicit homophobia and discrimination	Being listened to, ‘held’, and supported
	Ruptures in alliances, being passed on	Anticipating or fearing stigma and discrimination	Wanting sexuality to be acknowledged
	Queer acceptance and empowerment	Not wanting to approach	Being supported by LGBTQ+ peers and allies

#### 4.3.1 What are sexual minority women’s experiences of accessing specialist addiction treatment?

##### 4.3.1.1 Experiencing inadequate care

By definition, all of the study participants were people who might have benefited from attending a specialist addiction treatment service. Although only five participants had ever had any contact with an addiction service, the experiences of four of those were generally negative. The care provided was often felt by participants to be appropriate or unsuitable, or even potentially worsening their addiction problems.

One participant described multiple ways in which the first addiction service that she accessed was inadequate for her needs:

*“I went to the [name of addiction service], I didn’t stay, I hated it, they were not queer-inclusive, very insensitive, lack of knowledge, lack of understanding, lack of awareness, it was like…[pause] I felt like a statistic, a box they needed to tick off, if I’m honest I felt like if I stay here, I might as well just be drinking again.” – P20.*

For this participant, there were multiple dimensions to inadequate care, both specific to her sexual minority identity (lack of queer inclusivity) and more generalised (feeling dehumanised within the treatment process). The service did not explicitly reject her, but nevertheless communicated prejudice and erasure. In failing to recognise her, it created the idea that her continued engagement with the service would be harmful, functionally equivalent to “drinking again.” The sense of ‘lack of understanding’ or ‘lack of knowledge’ was echoed by two other participants, who felt the advice and support they were given within treatment services around alcohol dependence was too basic to be helpful to them.

A third participant spoke of the addiction service she attended as failing to help her, and even worsening her problems, despite engaging in treatment for alcohol dependence for a year:

*“It didn’t get better at all, that was when I started taking drugs as well.” – P7.*

Part of ‘inadequate care’ may involve services not understanding or acknowledging the ‘why’ behind an addiction problem for sexual minority women. For participant P7, drug and alcohol problems were partly due to having grown up in a repressive religious environment and having undergone forced gay conversion ‘therapy’, but this was not spoken about during her year of treatment. Attempting to address her presenting addiction problem for a year without engaging with the underlying identity-related stress and trauma suggests a structural inability of the treatment model to accommodate this type of history, and likely goes beyond a lack of practitioner knowledge: most UK addiction services are not designed or resourced to offer intensive trauma-focused psychological support.

##### 4.3.1.2 Ruptures in alliances, being passed on

Several participants spoke of feeling like they were ‘falling between the cracks’ of services (including addiction and general mental health services) or being passed between different organisations and health professionals. This was experienced as counter-therapeutic, repeatedly rupturing tentative alliances between patient and professional. Participant P7, who experienced an exacerbation in her addiction problems during treatment as above, linked this to having developed a relationship with the health professional she initially saw at an addiction service, enabling her to feel comfortable disclosing her past childhood abuse. However, this then led to her perception of being ‘passed on’ twice to a different professional during her treatment:

*“P7: I saw the same person. And then some stuff came out about the abuse. So I got moved on to a different person who was more qualified. Yeah.*

*MH: And was that sort of a counsellor or psychologist or something, or a key worker with more experience?*

*P7: There was, there were counsellors that were there. But then when I finished like the other person that I moved to, they said that again, that my issues was too much for that centre to deal with. And then I was referred to a psychologist. Who wasn’t like a drug and alcohol psychologist, just a psychologist.”*

Here, P7’s act of vulnerability—disclosing abuse to a person she trusted—was met with repeated institutional rejections (being passed to a stranger) rather than therapeutic containment. In conjunction with the previous theme, it is possible that her service experiences may have reproduced, in an attenuated form, the dynamics of a repressive religious upbringing or forced conversion ‘therapy’ itself: being in an institutional setting where authority figures are claiming to help you, but cannot be trusted and do not fundamentally see your identity; or of disclosures about who you are leading to relational rupture and rejection.

The sense of being ‘passed on’ was reflected at the service level in a participant’s experience of being shuffled between a mental health organisation to an alcohol service and back again after disclosing her drinking, being told she was not eligible for either in the process:

*“I did have quite an intense period of psychotherapy for about two years. […]. But when the therapist kind of talked a little bit about my alcohol usage, they said I wasn’t, I wasn’t, like, eligible to kind of be accessing their service because of the amount of alcohol that I drunk. So then I got referred to the alcohol service locally, and they said I didn’t meet their criteria. I didn’t drink enough to meet their criteria, I wasn’t getting up in the morning and drinking. So they referred me back to the, yeah, so it’s one of those. Yeah, I suppose psychiatry or whatever.” – P6.*

Here, bureaucratic institutional mechanisms imposed an artificial separation between ‘mental health’ and ‘addiction’ problems, and resulted in a de-legitimisation of both in the process: her drinking alcohol was seen as a contraindication to her mental health treatment, but was then deemed insufficiently problematic to be addressed by an addiction service. Although there may have been valid clinical reasons for being ‘passed on’, for both participants, these experiences led to a sense of fatalism or hopelessness around addiction treatment. Even when *trying* to seek help, participants felt rejected by the services or professionals they saw, following vulnerable disclosures. These experiences also meant that they were put off from seeking other avenues of addiction support. Participant P6 reported that even though she had been redirected to AA, she would have felt “silly” trying to attend after an alcohol service had effectively told her that her problems were not ‘serious enough’.

##### 4.3.1.3 Queer acceptance and empowerment

One participant reported a more positive experience of accessing specialist addiction support. They had been referred to attend drug treatment following arrest due to violent behaviour. This proved to be a place they felt accepted in their sexuality and gender identity, and where they found the staff to be very knowledgeable about LGBTQ+ issues. Although they took over a year of engaging in treatment to recover from drug dependence, they did eventually manage to do so, and felt this to be a significant achievement.

Despite having had generally bad experiences accessing or attempting to access several different sources of specialist addiction treatment, another participant did eventually find an integrated addiction and homelessness support service, which enabled her to recover from her drug and alcohol problems. The importance of services affirming minority sexualities and gender identities, and demonstrating knowledge, was echoed in her account of the service:

*“I actually had to do research before I found a new place to go to. Um, it’s called [name of service], it’s heavily queer-run, queer-inclusive, queer-minded. It’s like it caters for the queer individual trying to put their life back together piece by piece and it’s like, they didn’t just focus on like your addiction or what you’re struggling with, and it’s for both the drugs and alcohol, painkillers and all, it’s like we wanna make sure that what brought you there, even if you face it in the real world, it will never bring you back to this low point again. It’s like they acknowledge the fact that we know that you’re… that there’s a very… there’s a baseline of stress that will always exist in your life based on the fact that you probably have the sexuality or gender identity that the world is never gonna accept, and we’re not gonna pretend like it’s not gonna be there because what, what happens if one day you’re ostracised or you have a homophobic comment thrown at you and that triggers... we’re gonna make sure that you are ok, and thrive actually. I think that’s the word I’d use, ‘thrive’.” – P20.*

This participant describes a service that seems to have essentially operationalised minority stress theory as a treatment model: naming the presence of external stressors (e.g., being ostracised, homophobic comments) that are potential sources of harm, working against the internalisation processes that minority stress produces (e.g., affirming sexuality as central to both vulnerability and recovery), and itself functioning as a resource of community connection (“queer-run, queer-inclusive, queer-minded”). By showing understanding of (for example) the effects of homophobia, the service was able to engage and support this participant to not only recover from drug and alcohol addiction, but to begin to ‘thrive’ in the world outside the service. This holistic focus beyond just addiction problems—attending to the reasons underlying it and how those might be linked to future relapse triggers—stands in stark contrast to the experience of participant P7, who spent a year in treatment without her history of forced conversion ‘therapy’ being discussed.

#### 4.3.2 What are the barriers to accessing or remaining engaged in addiction treatment?

##### 4.3.2.1 Explicit homophobia and discrimination

The vast majority of participants in the study had not accessed addiction treatment services, and even some of those who had felt that they had not accessed services for long enough, or at the right times, to help them recover from their addiction problems. Explicitly homophobic experiences were a part of this, for example as participant P10 describes:

*“I think that access to [drug and alcohol] services and GPs and things was massively affected by my sexuality. […] My sexuality was a big issue and they like constantly, constantly went on and on about my sexuality and were trying to make me normal it seemed. So I think it was related because it didn’t matter what I did, I was wrong because my sexuality was wrong. Therefore everything about me was wrong.” – P10.*

Participants also described homophobia as a barrier to being able to openly speak about their experiences with addiction or mental health problems in services. This could take several forms: for one older participant, she was worried that disclosing she was a lesbian would lead to her losing custody of her children, for another, it meant that she ‘bottled up’ her emotions and did not speak about relevant experiences that might have been therapeutic to discuss in addiction treatment.

Other forms of discrimination could also function as barriers to remaining engaged in addiction treatment, as with this participant who rapidly left the first addiction service she tried to attend:

*“Sometimes I think part of the issue is that she was white [friend who attended the same addiction service and had a good outcome] and I was Black. I feel like they were nicer to white people, with people that weren’t white it was like the stereotype of the ethnic troublemaker who just needed… they wanted a quick fix and then it was like, ‘lock the door, go away’. They were just so impatient, so angry, like a parent berating you for being there in the first place.” – P20.*

Here, participant P20 explicitly performs an intersectional analysis of her service experience, which she felt produced different treatment outcomes along racial lines. The service did not respond to her as an individual seeking help, but as a racialised category that carried pre-existing assumptions about her motivations, character, and deservingness of care. The punitive and paternalistic character of this interaction, “like a parent berating you”, is produced by the intersection of racist stereotypes with institutional authority. Black women, in particular, may be subjects of ‘controlling images’ (66)—such as the ‘Jezebel’ figure or the ‘Angry Black Woman’ (67), socially constructed to justify their oppression. In this case, participant P20 was seen by the service as a problem to be managed and removed, in contrast with the “nicer” treatment her white friend received. For several participants, experiences of discrimination within healthcare services (both addiction services, and wider healthcare settings such as mental health and primary care) led to them fearing or losing trust in the services that they had hoped might be able to help them.

##### 4.3.2.2 Anticipating or fearing stigma and discrimination

Even if participants had thought addiction services might have been helpful, they feared or anticipated stigma from them, which could prevent them from even trying to approach services. This participant describes why she did not seek medical support for alcohol dependence:

*“I would have been very nervous about a service… because I would have been like, well, what if they’re homophobic to me, like everyone else in life has been? Like what, you know, and how, would they have understood the various stuff I was talking about, the reasons why I drank and the experiences I had, if they didn’t know anything about that world?” – P15.*

As this participant describes, previous more generalised experiences of homophobic or transphobic stigma may ‘prime’ anticipation of rejection, or of not being ‘understood’ if she were to approach services. She further explains:

*“I think also, like, if you’re LGBT, rejections often feel like they’re about that, even, if they come from straight people. Because you’re so used to that dynamic. And I don’t think straight people necessarily understand. But like, everything they are doing with you, they represent straight people as a group who have probably not been tremendously amenable to your interests.” – P15.*

For this participant, discrete homophobic experiences had produced a persistent interpretive framework through which subsequent interactions with the heterosexual world were filtered. Although this heuristic may have been self-protective, it stopped her from accessing help, which would have required her to trust representatives of the group from which prior harm has come.

This fear of stigma sometimes intersected with or was intensified by other minority identities, such having a minority racial/ethnic identity. For some participants from racial/ethnic minority backgrounds, both drug/alcohol use and non-heterosexuality were very highly censured in their communities, and they feared that seeking support might have led to these being disclosed. When explaining why she struggled to seek support for alcohol problems, participant P3 felt that it could have led to her wider family being shamed if others from her community had found out she was doing so:

*“I don’t want them to think, Oh she’s a drunk as well as her sexuality as well. I just don’t want it to be common knowledge… it wasn’t just how they were going to perceive me, because how they were going to judge the family as a whole. And my mum was like, I don’t care, but I said, I do.” – P3.*

Although the barrier to accessing addiction support (anticipation of stigma) was the same for both participant P15 and P3, the mechanisms through which this was operating are different. For participant P3, the reputation at stake was “the family as a whole” and not just her own. Participant P15 feared a homophobic service, whereas participant P3 feared her own community, and the consequences of being known to have sought help at all. This is a culturally specific dynamic that intersects with, but is not reducible to, either homophobia or addiction-related stigma. Her self-imposed concealment (protecting her family even when her mother says she does not need protecting) produced a form of anticipatory labour, i.e., managing the potential communal consequences of disclosure of multiple stigmatised identities.

##### 4.3.2.3 Not wanting to approach

With any drug or alcohol problem, accessing services usually requires that a person perceives there to be a problem, has a belief that the problem can be helped, and has knowledge of a place or service from which help may be offered (68). There are multiple reasons why sexual minority women may be less likely than heterosexual or cisgender women to have these beliefs and perceptions. One such reason may be that given the higher prevalence of drug/alcohol use within the community of sexual minority women, use for any one person may be seen as comparatively unproblematic, because others around the person are using similar or larger quantities of drugs/alcohol. For example, when describing why she did not seek addiction services, one participant explained:

*“But I think one of the main reasons I didn’t is because firstly, there wasn’t… I felt like there needed to be one particular drug that I was hooked on for it to be valid. And then secondly one of my friends was a lot worse than me, and I was like, Okay well, she’s not getting any help for it then I won’t either.” - P16.*

This was echoed by another participant, whose friend, a “heavy drinker,” discouraged her from perceiving her alcohol use was problematic:

*“Sometimes I would tell my friend I was like, Oh I think I’m drinking too much. And they were like, No you’re not, you’re fine. You know what I mean? And it was hard to see that’s too much.” – P4.*

Not realising that alcohol or drug use had become problematic could also dovetail with imagining that addiction services were designed for people with a higher level of need, who had stopped ‘functioning’ by society’s standards. Drug and alcohol services might be perceived to be only for “people with serious heroin addictions who have become homeless” (P1), and not for people who were managing to maintain employment or housing. Stereotypes about the ‘type of people’ who have addiction problems, and are therefore the target of addiction services, could also be gendered. Several participants discussed feeling that services were designed for men, and that they therefore did not see themselves needing them, as explained by this participant:

*“I thought of problem drinking as very much like a thing that, you know, your idea of an alcoholic is like a 50-year-old man in a park drinking out of a bag like, and that’s like, definitely, that’s awful, those people need services. But I didn’t see myself in that demographic.” – P15.*

The image of the “alcoholic”—in this case gendered, aged, publicly visible, and implicitly classed, protected the category of ‘addict’ from being applied to this participant, who was still at secondary school when her drinking problems started. The theme of ‘not wanting to approach’ services encompassed denial or non-recognition that drug or alcohol use had become problematic, and not wanting to or not knowing that they could seek help from services.

#### 4.3.3 What are the facilitators to accessing or remaining engaged in addiction treatment?

##### 4.3.3.1 Being listened to, ‘held’, and supported

Interview participants who had not accessed addiction services were asked to describe or imagine what would have helped them to do so. For those who had accessed services, they were asked what would have made those experiences better or more helpful. One participant, who had had negative experiences with addiction services as well as wider medical and social care services, put it succinctly:

*“MH: What would good support have looked like for you at a time when you might have needed it?*

*P10: Well I think being listened to, being believed, would have been great.”*

This sense of wanting to be ‘listened to’ and ‘believed’ often related to women’s desire to have their experiences and histories of trauma, abuse, and stigma acknowledged and understood. As this participant explained:

*“People use alcohol and drugs because of all the bigotry and homophobia and transphobia and whatnot, um, you really want someone you’re talking with, to empathise with that, and to understand, and not to have to revisit the trauma of like, explaining the homophobia and transphobia to someone who doesn’t understand it.” – P17.*

Healthcare encounters like the one described by participant P17, when sexual minority women are required to educate their healthcare professionals, place an additional burden on the person seeking help. As this participant describes, such education potentially acts as a form of re-traumatisation, compounding the negative impact of the original experience.

Women wanted to feel a sense of being emotionally and practically ‘held’—contained and supported by a service and its professionals, that could act as a place of safety during difficult periods of addiction. This was illustrated by one participant, who describes an experience shortly after leaving a residential addiction service:

*“Um, a few weeks later I actually ran into the person who assaulted me. And that was rough. And ‘normal me’ would have probably gone on a fender bender. But I remember hightailing it there [to the service], crying, saying what happened and I just said, look, I’m here because I’m not gonna take a pill, I’m here because I’m not gonna drink, I’m here because I’m gonna get better, I’m here because he has no power over me and I just… repeating it like some weird mantra. And I did calm down. And I remember [name of female service worker], she just held me, telling me you did good, love, you did good.” – P20.*

The above quotation speaks to a very concrete and physical example of being held and supported, but other participants described similar psychological approaches and attitudes that they would want from services. When asked to describe what ‘ideal’ support might look like within an addiction service, one participant explained:

*“Yeah, I guess again, it’s ‘you’re alright as you are’. Like when I think about the spaces where I have felt supported and where I have felt like I could do any fucking thing right now, it has always been when someone is celebrating you for how you are right now. […] Like, you are okay as you, and you deserve to be loved as you. Those are the spaces that make me feel like I’m safe.” – P12.*

‘Meeting the person where they are at’ in terms of drug/alcohol use has long been a principle of person-centred addiction care, but unconditional acceptance should also extend to a person’s sexuality and gender identity/presentation within services.

##### 4.3.3.2 Wanting sexuality to be acknowledged

Almost universally, participants wanted addiction services to identify, acknowledge, or address their sexuality. For people who had sought support around addiction from services of any kind (including primary care and mental health services), this was rarely asked about or brought up. One participant explained:

*“It’s about your identity isn’t it, you know? So you do think that there needs to be more with regards to that.” – P7.*

For some participants, it was important that services ask proactively about sexuality, because they may have felt uncomfortable or uncertain bringing it up spontaneously themselves. By asking the question, services could demonstrate that they were a place where sexuality could be openly talked about, and where it would be accepted as just another facet of identity.

Participants also spoke of the importance of services understanding the intersection of their identity as sexual minority women and their drug/alcohol problems. When asked about the importance of staff addressing LGBTQ+ identity, one participant responded:

*“Yeah, I think that’s quite key. I do because… because it’s who I am. And it has been, you know, I think going back to the past, those kind of sexuality issues did trigger a lot of my drinking. They don’t now, but they did then. So it is relevant. It’s very relevant.” - P6.*

Without being identified as such within services, opportunities may be missed to explore the context of sexual minority women’s drug/alcohol problems, and possibly the ‘why’ underlying them. However, it is also important for services not to ‘force’ disclosures of minority sexuality or gender identity. A few participants spoke to the experience of not initially trusting services, professionals, or support organisations enough to disclose this information.

##### 4.3.3.3 Being supported by LGBTQ+ peers and allies

Previous research has pointed to the value of peer support within addiction care for sexual minority people (69, 70). Participants felt that seeing a professional who is openly LGBTQ+, or explicitly an LGBTQ+ ally, could enhance relational trust and disclosure, contributing to a sense that they were understood and ‘seen’. As this participant explains:

*“I feel like it’s easier to, like, talk to someone who sometimes, on some primal level, like, the biggest part of your identity, knows what it’s like to be you and that will go a long way.” – P20.*

Several participants had specifically sought support around addiction from professionals that they perceived to be non-judgemental, allies, or LGBTQ+ friendly (or who were known to be LGBTQ+ themselves). As people attending statutory addiction treatment services typically do not have a choice about the professionals they are assigned, they had often sought out private counselling or psychotherapy to help them with addiction problems. Here, one participant explains the benefit of having a therapist who also has a minority sexuality and gender identity:

*“…my therapist for the last few years is non-binary and so it’s so nice to, as I’ve said, I’ve had therapy for most of my life, I’ve had a lot of different therapists, but they are the first person that’s been queer, and it’s so much easier to talk to them about stuff. I don’t have to explain things, the number of times I’ve had to explain to so-called professionals what being gay is or how being trans affects my life. It just turns an hour-long session into 5 minutes of talking about myself and 55 minutes explaining the history of transgenderism. Whereas with them I can just walk in and be like, I feel really dysphoric today and they go, Yeah I got you, I get it.” – P5.*

Seeing an LGBTQ+ peer/professional allowed women to ‘get to the heart of the matter’ more quickly, not having to explain themselves or their identities. For this participant, there is a quantified sense of therapeutic loss: in the very spaces designed to help her psychologically, she has had to spend most of her time performing the labour of making the realities of her life intelligible. With her current therapist, being immediately legible contributed to a sense of relational safety. This may be particularly important for sexual minority women who are not cisgender, for whom multiple intersecting axes of marginalisation collide.

## 5 Discussion

This study examined sexual minority women’s experiences of accessing (or not accessing) addiction treatment services in England. Taken together, our nine themes highlight how structural and interpersonal dynamics shape the addiction treatment trajectories of sexual minority women, reinforcing previous evidence that they encounter unique barriers when engaging with addiction healthcare (69, 71, 72). Our findings are situated within the normative framework of minority stress theory, but we have also sought to pay attention to institutional factors, and to draw out examples where intersectional understandings are key. For the majority of participants in our study, addiction services had not been approached at all. When they were, care could be inadequate, fragmented, or stigmatising. In some cases, treatment services served as sites of minority stress production, rather than amelioration, with recovery occurring despite the service system, rather than because of it. Previous negative experiences of health and social care will almost inevitably produce a reluctance to seek or engage in further care (73–75), and here, they prevented access to the conditions that sexual minority women identified as necessary for addiction recovery. Participants in our study also identified key facilitators for service access and engagement, such as feeling genuinely listened to and supported, having their sexual identity acknowledged and accepted in treatment, and receiving support from LGBTQ+ peers or allies. These findings contribute to a growing body of qualitative research demonstrating that identity-affirming and relational approaches are central to effective healthcare for sexual minority women (76, 77). Here, we have demonstrated that they extend to addiction-related healthcare, which is relatively novel in the literature.

The accounts of participants who did not access services suggest that ‘addiction’ itself may be operating not as a neutral category, but as a culturally constructed identity that must be claimed (or imposed) before help-seeking becomes possible (78). The biomedical framing of addiction, or ‘disease model’ (79) sees it as a chronic condition underlain by structural and functional brain changes, operating independently of social identity. However, this model has been developed primarily through research on male, publicly visible, ‘dysfunctional’, and often criminalised populations, which has affected its diagnostic criteria, treatment models, and public representations (80–84). When sexual minority women do not see themselves in these representations (for example, because their drug and alcohol use is socially and subculturally embedded, involves polysubstance use, serves useful functions, or is simply female and queer)—the category fails to help them. Furthermore, it renders their particular relationship with drugs and alcohol institutionally invisible. The clinical framing that sees sexual minority women as being ‘in denial’ about their addiction problems (85) may be inadequate: this can be seen as a rational response to a category that has been constructed in terms that exclude them.

Participants’ accounts of inadequate or insensitive care reflect longstanding critiques that healthcare systems are structured around heteronormative and cisnormative assumptions (86–88). Addiction services are not immune to this, despite being places where LGBTQ+ people may routinely present or be over-represented (89). Such assumptions privilege the experiences of heterosexual and cisgender people, and can render sexual minority women’s needs invisible, leading to invalidating healthcare experiences when they do not fit the implicit templates of ‘expected’ patient identities. This aligns with Dawson et al.’s work (76), in which sexual minority women reported that a “dominant culture-centric” (i.e., heteronormative) system and insufficient provider knowledge created barriers to accessing care. Our participants’ descriptions of services lacking understanding of LGBTQ+ lived realities mirror findings that addiction healthcare professionals often receive little training in LGBTQ+-specific issues (90), potentially resulting in misrecognition or minimisation of minority stress or trauma histories. Normative assumptions within addiction services can also produce barriers to sexual orientation disclosure (91), and previous research has indicated that discussions around LGBTQ+ identity were rarely invited by service providers (54). Identity concealment contributes to the minority stress experienced by sexual minority women, and may have significant negative psychological consequences (16). Minority identity may be something that people only feel comfortable discussing in a treatment context once they have built up confidence that this will be handled sensitively.

Attitudes of addiction health professionals towards sexual minority people may range from affirming to starkly prejudicial (92, 93), with the latter potentially accounting for the experiences of direct homophobia and discrimination that were reported. Such experiences are previously well-documented for sexual minority women seeking addiction care (69, 77, 94, 95). Infantilising or paternalistic consultation styles (which may also have a racialised element) towards sexual minority women have also been previously reported (75, 96). Explicit homophobia and implicit discrimination within treatment environments reflects the ways institutional cultures construct normative assumptions about sexuality, positioning LGBTQ+ identities as deviant, pathological, or irrelevant to treatment (97, 98). When services reproduce these assumptions in practice, they not only marginalise sexual minority women but reify clinical or commercial narratives in which addiction is framed as a ‘personal responsibility’ without reference to wider contexts (99, 100), despite strong evidence that sexual minority women’s drug/alcohol use is shaped by identity-related stress, trauma, and social factors (49, 72, 101, 102). There is evidence that structural interventions, such as specific LGBT training for health professionals, can improve attitudes, knowledge and skills (103), and this may help reduce discriminatory healthcare experiences for sexual minority women.

Even when not directly experienced, participants anticipated hostility or judgement due to their sexuality or gender identity, a feeling grounded in wider social experiences of prejudice. Consistent with this, Murray et al. found that LGBTQ+ individuals often prefer to seek informal support or no support at all rather than risk exposure to prejudice in formal addiction services, which are perceived to cater to a heterosexual male ‘norm’ (104). People may self-exclude from care because they expect (based on lived realities) that they will face intersecting stigmas: as a sexual minority person, a woman, and someone with an addiction problem (105, 106). This can lead to compounded shame and hesitancy to approach services. Sexual minority women’s reluctance to seek help can thus be seen as a rational response to a health system that has historically marginalised them. Community norms that lead sexual minority women to underestimate the severity of their drug/alcohol problems, and the need to seek help for them, may compound this. Some preliminary research that suggests culturally tailored psychoeducation reduces alcohol-related risks among lesbian, bisexual and queer women (107), although there are few existing psychoeducation resources aimed specifically at addressing drug/alcohol problems among this group.

Participants’ accounts of being passed between services and professionals reflect institutional processes that classify service users into pre-existing categories (e.g., mental health vs. addiction patient) despite sexual minority women’s lives frequently cutting across these boundaries. This is likely to be illustrative of wider political decisions: in 2012, the UK Government’s Health and Social Care Act transferred responsibility for commissioning addiction (but not mental health) services from the NHS to local authorities (108), and addiction services have subsequently seen significant reductions in real-term funding (109), forcing many to tighten eligibility criteria. This structural separation is likely to most significantly impact people whose drug and alcohol use is deeply entangled with other dimensions of their experience (e.g., identity-related trauma, minority stress, or the social organisation of a subcultural community life around drug-taking/drinking). For sexual minority women, the institutional insistence on sorting them into a ‘mental health’ or an ‘addiction’ box produces the experience of ‘falling between the cracks’ that the participants describe.

As Berger and Luckmann note, institutional classifications shape what kinds of identities and problems are considered thinkable and treatable (97). When services operate along rigid eligibility protocols, sexual minority women with complex, intersecting needs risk being rendered ‘out of place’ within any one setting. The resulting circulation between professionals and services may not only fragment, delay, and disrupt treatment, but can reinforce women’s perceptions that their identities and needs do not fit legitimised biomedical models of addiction or mental illness (100, 110). Holistic models of care that can simultaneously address (for example) mental illness, addiction, and the effects of gender-based or homophobic violence, may facilitate access and engagement (77, 94, 111).

Despite these barriers, we also highlight what can facilitate sexual minority women’s access to, and retention in, addiction treatment. Participants essentially wanted services that are affirming, inclusive, and supportive, aligning with broader calls in public health to tailor interventions for sexual minority women and improve the ‘cultural competence’ or ‘cultural humility’ of services (72). Previous research indicates that simple measures can make a difference: displaying rainbow flags or posters, offering inclusive intake forms, and explicitly mentioning LGBTQ+ support can help services indicate an affirming stance (77, 99, 112, 113). Genuinely welcoming environments can mitigate minority stress; indeed, affirming care can reduce treatment barriers and improve outcomes by buffering patients from the stigma and stress they face elsewhere. Services and the professionals within them using a relational, person-centred, trauma-informed approach improves outcomes in addiction recovery (114, 115). Foregrounding safety, choice, collaboration and empowerment is important for marginalised groups in general, and particularly for sexual minority women, to build the trust that is a prerequisite for service engagement (116). Service encounters are not passive events: relational validation also helps construct a recovery identity rooted in dignity and self-worth (117), shaping how sexual minority women come to understand themselves as deserving of care. This may be facilitated though employing LGBTQ+ peer supporters or health professionals, with whom sexual minority women may feel more at ease. There is evidence that this is helpful for sexual minority men experiencing problems with chemsex (118), and for people experiencing addiction problems in general (119). LGBTQ+ communities have historically served as sites of mutual aid and collective action (120), and peer interactions may help constitute recovery identities grounded in community rather than isolation. Our findings suggest that services have a complex task: not simply to ‘ask about sexuality’, but to create conditions of sustained trust, safety, and demonstrated LGBTQ+ competence under which sexual minority women feel genuinely and meaningfully seen.

## 6 Strengths and limitations

The use of reflexive thematic analysis provided a transparent framework for coding and theme development, emphasising researcher positionality and reflexivity to produce trustworthy qualitative insights. Sample diversity was another strength: the 21 participants encompassed a range of sexual orientations (e.g., bisexual, lesbian, queer) and gender identities (e.g., cisgender and trans women), enriching the data with varied perspectives. Approximately one-third of the participants belonged to racial/ethnic minority backgrounds, and although this is reflective of the wider population of England (81.7% of the population of England and Wales identified as white in the 2021 Census (121)), it means that our findings are disproportionately shaped by the experiences of white women. This may have led to an analysis where sexuality and gender identity, not race, are seen as the primary axes of marginalisation within service encounters, and where recommended facilitators to service access fail to benefit racially minoritised women. In addition, the sample was relatively young, with only four participants over 40 years of age. Changing patterns of LGBTQ+ social acceptance may mean, for example, that older women may have navigated services during greater periods of institutional homophobia, and future research should investigate the experiences of middle-aged and older sexual minority women. Recruitment via social media and LGBTQ+ groups/mailing lists may also have selected for participants who are digitally connected, sufficiently ‘out’ to respond to LGBTQ+-targeted recruitment, and who have the literacy and confidence to engage with an online research invitation. This may have excluded potential participants who are closeted, or experiencing more extreme marginalisation. It also may have attracted participants who are already engaged with typical LGBTQ+ community discourses, and so understand and describe their experiences through the lens of (for example) minority stress.

MH’s lived experience as a sexual minority woman offered valuable insider insight, allowing rapport with the interviewees and grounding the interpretation of findings in a more authentic understanding. This potentially facilitated nuanced data collection and analysis, that ‘outsider’ researchers might overlook. However, the reflexive approach of the study acknowledges that the research team’s positioning may affect the way that interviews are conducted, data interpreted, or findings presented. For example, as a mostly LGBTQ+-identifying team, we may have been more likely to interpret negative addiction service experiences through the lens of identity-related stigma, rather than the products of an underfunded, overstretched system that frequently treats people “like a statistic” regardless of sexuality. The clinical experience of MH and members of the wider research team means that we were professionally embedded in the biomedical and institutional frameworks under investigation, which may have limited how far our critiques extend. Our clinical positioning also implicitly assumes that ‘improved access to addiction services’ is a desirable outcome, which may not always be the case for sexual minority women, if these are sites of minority stress production or re-traumatisation. Our findings are specific to the addiction service landscape in England, and we do not claim generalisability of our findings beyond similar contexts, rather aiming to explore our participants’ experiences in detail. We also did not seek feedback from the participants about our findings or the interpretation of their interviews, which may have strengthened our conclusions.

## 7 Conclusion and recommendations for practice and policy

Explicit discrimination and stigma, or justified fear of this, shapes sexual minority women’s experiences of accessing/not accessing addiction services. Belonging to multiple marginalised groups, sexual minority women often find it hard to find addiction support that meets their needs, or is appropriately tailored to them. Participants in this study broadly wanted to access services that were affirmative of their sexualities and gender identities, and knowledgeable about the effects of trauma and discrimination, whilst not assuming that their identities were the cause of their addiction problems. Sexual minority women perceived that statutory addiction services, or individual health professionals working there, lacked the ‘cultural competence’ around LGBTQ+ health needed to sensitively engage with them.

For services, specific adaptations to existing service environments, as well as commissioning new service models, may improve sexual minority women’s experiences (37). Existing services can develop and implement non-discrimination policies, provide staff training on LGBTQ+ healthcare, and include visible signs of LGBTQ+ affirmation such as positive waiting-room literature or adverts for local LGBTQ+ support groups. Providing separate spaces for women and people who experience gender-based violence, and offering both online and in-person treatment (e.g., groups for addiction recovery) may facilitate access for women with histories of trauma. Employing sexual minority and/or female peer supporters and professionals to facilitate referrals into and engagement within addiction treatment services may be beneficial, increasing the sense of trust or ‘therapeutic alliance’ between a service and sexual minority women patients. Services could also consider targeted interventions such as outreach events in LGBTQ+ community and social spaces, or providing information specifically addressing the barriers/facilitators experienced by sexual minority women. New service models might include services run by and for LGBTQ+ people, or integrating both sexual health and addiction support (55, 122, 123). By listening to sexual minority women’s voices and incorporating their recommendations, we can begin to dismantle the barriers to care and enable equitable access to addiction recovery for this often-overlooked population.

## Data Availability

The datasets presented in this article are not readily available because of the nature of the interviews conducted for this study. Due to the potential for identification of participants, we have made the decision not to share or upload any transcripts. Requests to access the datasets should be directed to Miriam Hillyard, miriam.hillyard@kcl.ac.uk.

## Footnotes

Edited by: Paolo Abondio, IRCCS Institute of Neurological Sciences of Bologna (ISNB), Italy
